# Differences in the Effectiveness of Long-Acting Injection and Orally Administered Antipsychotics in Reducing Rehospitalization among Patients with Schizophrenia Receiving Home Care Services

**DOI:** 10.3390/jcm8060823

**Published:** 2019-06-09

**Authors:** Hsiao-Fen Hsu, Chia-Chan Kao, Ti Lu, Jeremy C. Ying, Sheng-Yu Lee

**Affiliations:** 1 Department of Psychiatry, Kaohsiung Veterans General Hospital, Kaohsiung 81362, Taiwan; sflin@vghks.gov.tw (H.-F.H.); tlu@vghks.gov.tw (T.L.); 2 Department of Nursing, I-Shou University, Kaohsiung 82445, Taiwan; bettykao@isu.edu.tw; 3 School of Public Health and Management, Wenzhou Medical University, Wenzhou 325035, China; jying@wmu.edu.cn; 4 Department of Psychiatry, National Cheng Kung University Hospital, Tainan 70403, Taiwan; 5 Department of Psychiatry, Faculty of Medicine, Kaohsiung Medical University, Kaohsiung 807, Taiwan; 6 Department of Psychiatry, College of Medicine, National Yang-Ming University, Taipei 112, Taiwan

**Keywords:** schizophrenia, psychiatric home care service, rehospitalization, long-acting antipsychotics, oral antipsychotics

## Abstract

The current study explored the differences in the effectiveness of first and second generation long-acting injections and orally administered antipsychotics in reducing the rehospitalization rate among patients with schizophrenia receiving home care services in a medical center in Southern Taiwan. Longitudinal data between 1 January 2006, and 31 December 2015, were collected retrospectively. Patients were classified into three treatment groups: First generation antipsychotic (FGA) long-acting injection (LAI), second generation antipsychotic long-acting injection (SGA) (LAI), and oral antipsychotics. The primary outcomes were the rehospitalization rate and the follow-up time (duration of receiving home care services) until psychiatric rehospitalization. A total of 78 patients with schizophrenia were recruited. The average observation time was about 40 months. The oral treatment group tended to be older with a higher number of female patients and a lower level of education. The FGA treatment group tended to have a higher frequency and duration of hospitalization before receiving home care services. We found no significant differences in the follow-up time or psychiatric rehospitalization rate after receiving home care services among the three treatment groups. We propose that oral and LAI antipsychotics were equally effective when patients received home care services. Our results can serve as a reference for the choice of treatment for patients with schizophrenia in a home care program.

## 1. Introduction

Schizophrenia is a chronic and burdensome mental disorder, causing diverse disturbances in cognition, mood, and reality testing [1]. As a result, it has a major impact on social and occupational functions. Approximately half of all patients with schizophrenia fail to adhere to treatment plans, which results into an increased risk of relapse, self-harm, an increased personal burden, high healthcare expenses, and a progressive clinical decline [2,3]. Therefore, the prevention of relapse during the maintenance treatment of schizophrenia is important because relapses often lead to rehospitalization [4,5]. However, it has been reported that over 40% of schizophrenic patients were rehospitalized within two years of being discharged [6].

Poor adherence and non-adherence to treatment are common problems for patients with schizophrenia, ranging from 40–50%, leading to an increased relapse risk, rehospitalization, and poor outcomes [7]. In a one-year real-world study, non-adherence to medication increased the risk of relapse almost five-fold compared to patients continuing antipsychotic treatment [8]. The tolerability of antipsychotics was a critical issue [9]. Long-acting injectable (LAI) antipsychotic medications were developed to improve drug adherence, which have been shown to attenuate the risk of relapse and subsequent episodes of rehospitalization [10,11,12]. In addition, LAI also improved the quality of life of patients and provided neuroprotective effects [10,13,14]. Both first and second generation antipsychotics are available in long-acting injectable versions. Although second generation antipsychotics (SGA) were developed to lower the risk of extrapyramidal symptoms, compared to first generation antipsychotics (FGA), SGA medications were associated with causing weight gain and metabolic syndrome [15]. The efficacy between oral forms of SGA and FGA has been compared by many studies with inconclusive findings [16,17]; the most famous, the clinical antipsychotic trials of intervention effectiveness (CATIE) schizophrenia trial, showed that at moderate doses, SGAs were not superior in safety or effectiveness compared to FGA [18]. For the LAI form of SGA and FGA, a randomized, double-blind clinical trial showed no statistically significant difference in the rate of efficacy failures after about 24 months of follow-up [19]. Although LAI was introduced to improve adherence, one commonly used LAI form of SGA, Risperdone, did not differ significantly from its oral form in the risk of rehospitalization in schizophrenic patients in a randomized trial followed up for 2 years [6]. Therefore, the efficacy of SGA and FGA in LAI form in patients with schizophrenia remained undetermined. 

A home care service is a program of frequent home visits including symptom evaluation, medication management, and family psychoeducation provided by registered nurses and physicians. The visiting frequency ranges from once every week to once every month. Home care services in Taiwan are usually provided to patients with limited drug adherence in either outpatient clinics or patients that were just discharged from an acute psychiatric ward. Such a home care service might deliver LAI to the patient’s home to ensure better drug administration compared to an outpatient department. Home care services were reported to dramatically decrease in hospitalization and to increase patient satisfaction [20]. Compared to a traditional outpatient treatment program, home care services for patients with schizophrenia were found to result in improved treatment adherence, decreased clinical symptoms, increased social interaction, and less perceived stress by families after frequent home visits. As for the treatment modality, it was reported that LAI treatment could delay rehospitalization compared to oral medication for patients receiving home care services, according to a population-based health insurance database study in Taiwan after 12-month follow-up [21]. However, there were no differences observed in the rehospitalization rate between SGA LAI (Risperdone) and FGA LAI [21]. Studies with a longer follow-up time may be needed to clarify the effectiveness of the difference in FGA, SGA LAI, and oral medication in the rehospitalization rate in a home care program.

The purpose of this study was to explore the differences in the effectiveness of FGA, SGA LAI, and orally administered antipsychotics in reducing rehospitalization among patients with schizophrenia receiving home care services in a medical center in Southern Taiwan from a ten-year database. We assumed that a longer follow-up time might be able to determine the differences between the effectiveness of FGA, SGA LAI, and oral antipsychotics in schizophrenia.

## 2. Methods

The Institutional Review Board for the Protection of Human Subjects at Kaohsiung Veteran’s General Hospital approved this research protocol. The project identification code is VGHKS16-CT12-10, and the approval date was 18 November 2016.

### 2.1. Patient Selection

The study was based on a chart review. Longitudinal data between 1 January 2006, and 31 December 2015, were collected for patients diagnosed with schizophrenia who received home care services (International Classification of Diseases, Ninth Revision, Clinical Modification, (ICD-9-CM) code 295). 

The inclusion criteria were (1) diagnosis of schizophrenia, (2) over 20 years of age, and (3) referred to a home care service right after discharge from hospitalization in an acute psychiatric ward. Patients with any major mental illnesses besides schizophrenia, such as bipolar disorder, borderline personality disorder, substance use disorder, mental retardation, and cognitive disorders were excluded. Patients who died within the follow-up time frame were also excluded. The patients were classified into 3 treatment groups according to the treatment they received: First generation antipsychotic (FGA) long-acting injection (LAI), second generation antipsychotic (SGA) long-acting injection (LAI), and oral medication. The FGA group included patients receiving Haloperidone and Fluanxol depot injections. The SGA LAI group included patients who received Risperidone consta and Sustenna. Patients who did not receive any depot injections were classified into the oral medication group, which included oral forms of Olanzapine, Risperidone, Ziprasidone, and Aripripazole. 

We retrieved the demographic data from each patient and their medical history before and after receiving home care services. Because the major outcome of this study was the risk of psychiatric rehospitalization, we set the index date as the first day the patient received a psychiatric prescription from the home care service. 

### 2.2. Statistical Methods

The statistical software package SPSS (Version 18.0) (SPSS Inc, Chicago, IL, USA) was used to analyze the data and *p*-values less than 0.05 were considered statistically significant.

We adopted one-way analysis of variance (ANOVA) to analyze the numerical demographic data at the index date among the 3 treatment groups and chi-squared tests to analyze differences in categorical demographic variables.

The rate of psychiatric rehospitalization was estimated using the Cox proportional hazard model controlling for differences in demographic data including sex, age, years of education, and hospitalization times before receiving home care service.

## 3. Results

### Participant Characteristics

A total of 78 patients with schizophrenia were included. Initially, we identified 96 patients with a diagnosis of schizophrenia in the home care program. However, fifteen patients were excluded because they were not referred to a home care service right after discharge from hospitalization in an acute psychiatric ward. Another three patients were excluded since they died within the monitored time frame. The demographic characteristics of these patients are shown in Table 1. The three groups differed in sex, age, educational level, and time and days of hospitalization before home care service. The oral group was found to be older, with more female patients and a lower educational level. There were no significant differences in onset age, marital status, and employment among the three treatment groups. The FGA group seemed to have greater hospitalization frequency and duration before receiving home care service (Table 1). We found no significant differences in the follow-up time or the psychiatric rehospitalization rate after receiving home care services among the three treatment groups (Table 2).

The effects of a different treatment on rehospitalization are shown in Table 3. We found no significant difference in the risk of psychiatric rehospitalization in the FGA and SGA treatment groups compared to the oral treatment group after checking for sex, age, years of education, and hospitalization times before receiving home care service.

**Figure 1:**
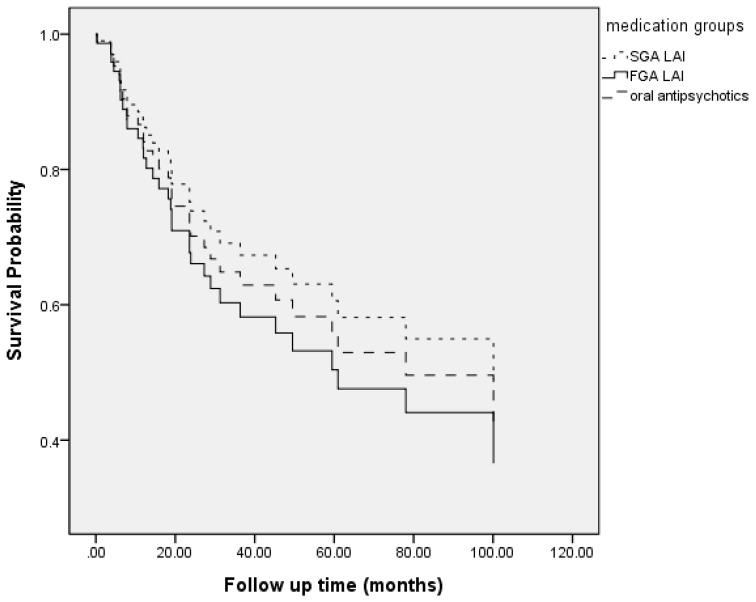
Follow-up time to psychiatric rehospitalization. (FGA LAI: first generation antipsychotics long acting injection; SGA LAI: second generation antipsychotics long acting injection.).

The survival curve of the risk of psychiatric rehospitalization in the three treatment groups is illustrated in Figure 1.

## 4. Discussion

With an observation period of over 40 months, our study reported that there were no significant differences in the risk of rehospitalization regardless of the treatment the schizophrenia patients received from home care services. Our study results agreed with McEvoy’s study [19] that reported that the use of SGA LAI did not significantly differ from the use of FGA LAI in efficacy failure (psychiatric rehospitalization) in a randomized clinical trial followed up to 24 months. Our study compared the differences among medications in a more practical clinical way, since the patients were not randomly assigned to each treatment group. Furthermore, our study included an oral medication group, which McEvoy’s [19] study did not. Although the sample size of the current study was limited, the rehospitalization rate at about 20+ months was about 0.6, which is similar to that with a 24 months follow-up time [19]. We therefore propose that medication itself might not affect the risk of rehospitalization for patients receiving home care services. However, we were unable to infer the effect of a home care service alone on the risk of psychiatric rehospitalization, since it was unethical for us to arrange another group that received home care services without taking any medication. We still propose that home care services alone might have some influence on treatment efficacy.

In the current study, patients in the oral medication group were mostly females at an older age and a lower educational level compared to the other two treatment groups. No significant differences in clinical characteristics were seen between the FGA and SGA LAI groups. Our data showed that age, sex, and education had no significant effect on the risk of psychiatric rehospitalization. The above factors also showed an inconclusive effect on drug adherence [22]. Since we found no significant difference in the risk of psychiatric rehospitalization between the oral and LAI groups, we assumed the patients in the oral group had a fair drug adherence. Our data demonstrated that the oral and LAI groups had a similar treatment efficacy. 

However, our study results differed from a study with a larger sample size using data from psychiatric inpatient medical claims data (PIMC) released by the National Health Research Institute in Taiwan [21], which found that patients treated with LAI had a significantly lower risk of rehospitalization than those treated with oral medication under a home care program within a 12-month follow-up period. Although that study [21] had a larger sample size, the follow-up time was shorter compared to ours, which spanned about 40 months. The rehospitalization rate during a 12-month observation period was about 30% in Ju’s study [21], while we found that about 16.7% of patients were rehospitalized during the first 12-month observation period. The lower hospitalization rate and smaller sample size of our study compared to that of Ju et al. might explain the differences between the two studies. We propose that a longer follow-up time with a larger sample size may provide more precise data about the treatment accuracy.

In the current study, the patients were not randomized to different treatment groups. The FGA LAI group had more psychiatric hospitalizations and a longer duration of psychiatric hospitalization before receiving home care services. It is possible that the FGA LAI group was more likely to be rehospitalized due to their frequent rate of hospitalization prior to entering the home care service, and therefore they may have had the most to gain from being put on LAI treatment. In real clinical practice, patients with poor drug adherence were more likely to receive LAI, and patients with fair drug adherence and better insight were more likely to be administered oral medication. From our study, we assumed that for patients with non-ideal drug adherence, LAI was as effective as oral medication. Therefore, LAI remained a good treatment option for those who could not adhere to oral medication. However, since this was a chart review study, we were unable to compare treatment outcomes in other aspects, such as psychotic symptoms, cognitive function, and/or occupational performance.

Nevertheless, our study had several limitations. First, our study consisted of less than 80 patients, which resulted in a limited statistical power. To obtain at least 80% power to detect a difference in the survival curve (two-sided logrank test, α = 0.05), assuming a hazard ratio at 0.98 [19], we would need a sample size of *n* = 298. However, we reviewed almost all patients with schizophrenia receiving home care services in the past 10 years in the case hospital. A larger sample size might require an even longer research span. Therefore, the current study results need to be interpreted with caution. In addition, the present study only used rehospitalization as a treatment outcome, lacking of other clinical parameters which might also demonstrate the treatment effect of schizophrenia, such as mood, cognitive symptoms, side effects, metabolic disturbances, and socio-occupational functions. Second, a future prospective study is warranted to demonstrate differences in the clinical aspects mentioned above between oral medication and LAI. In addition, SGA LAI usually costs more than FGA LAI. The precise cost effectiveness was not calculated. To conduct such an economic analysis, the health care cost data is needed, which was not available for the current study. Third, the current study did not include treatment groups such as patients receiving oral medication or LAI in an outpatient clinic setting and patients receiving only homecare services without medication. Such a design may better differentiate the influence of home care services and is warranted in a future study. In addition, the LAIs and oral medications were chosen because they were part of the drug formulary available in this medical center. A future study consisting of the more comprehensive available LAIs is needed. Fourth, we did not control other medication or other medical illnesses; therefore, our result should be interpreted with caution. Fifth, the current study only relied on a chart review and lacked matching between treatment groups. This influenced our outcomes and its application. A future study with matching treatment groups is needed. Finally, the current study is not a randomized double-blind design. Our study results may not reflect the real effectiveness of LAI versus oral medication.

In conclusion, we observed no differences in the risk of rehospitalization in patients with schizophrenia under oral medication or LAI receiving home care services after a 40-month observation period. We also found that neither sex, age, education, nor previous time of psychiatric hospitalization influenced the risk of psychiatric rehospitalization. Based on our findings, we propose that oral and both SGA and FGA LAI antipsychotics are equally effective when patients received home care services. However, these conclusions should be interpreted with caution since our sample size was too small to detect a difference. Future studies with larger populations are needed to reconfirm our results.

## Figures and Tables

**Table 1:** Basic characteristics of patients in different treatment groups.

	FGA Depot	SGA Depot	Oral Medication	F or χ^2^	*p*	Post Hoc
***n***	19	42	17			
**Sex (male/female) (*n*)**	9/10	20/22	2/15	7.1	0.029 *	SGA = FGA ≠ Oral
**Age (years)**	47.4 ± 11.9	47.9 ± 10.5	57.9 ± 17.2	4.6	0.013 *	FGA = SGA ≠ Oral
**Onset age (years)**	29.8 ± 8.9	32.5 ± 11.2	35.4 ± 14.4	1.0	0.36	
**Education (≤9 years, >9 years) (*n*)**	5/14	11/31	10/7	6.4	0.042 *	SGA = FGA > Oral
**Marital status (single/married divorced) (*n*)**	9/9/1	25/12/5	5/11/1	7.2	0.12	
**Job (yes/no) (*n*)**	1/18	7/35	1/16	2.35	0.31	
**Hospitalization times before home care service (*n*)**	5 ± 3.5	2.2 ± 1.5	2.1 ± 1.4	12.3	<0.001 *	SGA = Oral < FGA
**Hospitalization duration before home care service (months)**	7.1 ± 6.4	3.1 ± 2.5	2.4 ± 1.7	9.3	<0.001 *	SGA = Oral < FGA

FGA: first generation antipsychotics; SGA: second generation antipsychotics. * *p* < 0.05.

**Table 2:** Hospitalization data before and after entering home care services.

	FGA Depot	SGA Depot	Oral Medication	F or χ^2^	*p*	Post hoc
	*n* = 19	*n* = 42	*n* = 17			
Follow up period under home care service (months)	44.4 ± 39.4	37.8 ± 32.3	40.5 ± 34.4	0.24	0.79	
Rehospitalization rate after home service care (%)	36.8	47.6	35.3	1.06	0.59	

**Table 3:** The effect of a different treatment on rehospitalization using Cox regression.

	Exp(B)	95%CI	*p*
**Treatment Groups**			
**Oral medication**	1		
**FGA**	0.77	0.22–2.62	0.61
**SGA**	1.17	0.43–3.18	0.76
**Sex**	1.38	0.67–2.86	0.38
**Age**	0.98	0.95–1.02	0.30
**Education**	1.22	0.53–2.77	0.64
**Hospitalization times before home care service**	1.04	0.89–1.20	1.04

References groups are: Patients under oral medication, female, Education > 9 years; FGA: first generation antipsychotics; SGA: second generation antipsychotics. CI: confidence interval.
